# Oculomotor feature discrimination is cortically mediated

**DOI:** 10.3389/fnsys.2023.1251933

**Published:** 2023-10-12

**Authors:** Devin H. Kehoe, Mazyar Fallah

**Affiliations:** ^1^Department of Psychology, York University, Toronto, ON, Canada; ^2^Centre for Vision Research, York University, Toronto, ON, Canada; ^3^VISTA: Vision Science to Applications, York University, Toronto, ON, Canada; ^4^Canadian Action and Perception Network, Canada; ^5^Département de Neurosciences, Université de Montréal, Montréal, QC, Canada; ^6^College of Biological Science, University of Guelph, Guelph, ON, Canada

**Keywords:** visuomotor integration, visual features, feature guidance, feature discrimination, eye movements, target selection, saccade planning, behavioral relevance

## Abstract

Eye movements are often directed toward stimuli with specific features. Decades of neurophysiological research has determined that this behavior is subserved by a feature-reweighting of the neural activation encoding potential eye movements. Despite the considerable body of research examining feature-based target selection, no comprehensive theoretical account of the feature-reweighting mechanism has yet been proposed. Given that such a theory is fundamental to our understanding of the nature of oculomotor processing, we propose an oculomotor feature-reweighting mechanism here. We first summarize the considerable anatomical and functional evidence suggesting that oculomotor substrates that encode potential eye movements rely on the visual cortices for feature information. Next, we highlight the results from our recent behavioral experiments demonstrating that feature information manifests in the oculomotor system in order of featural complexity, regardless of whether the feature information is task-relevant. Based on the available evidence, we propose an oculomotor feature-reweighting mechanism whereby (1) visual information is projected into the oculomotor system only after a visual representation manifests in the highest stage of the cortical visual processing hierarchy necessary to represent the relevant features and (2) these dynamically recruited cortical module(s) then perform feature discrimination via shifting neural feature representations, while also maintaining parity between the feature representations in cortical and oculomotor substrates by dynamically reweighting oculomotor vectors. Finally, we discuss how our behavioral experiments may extend to other areas in vision science and its possible clinical applications.

## 1. Introduction

As your eyes scan through your sock drawer looking for your favorite blue socks, you redirect your gaze to each pair of blue socks one-by-one. Although you attempt to ignore all other colors, you occasionally find yourself distracted by the same pair of bright red socks, which repeatedly draw your gaze back to them. Anecdotes like this illustrate how visual features guide our voluntary and reflexive eye movements in our daily lives, a phenomenon also routinely observed in primate vision experiments. For example, when humans or monkeys perform goal-directed search for a previewed target, their eye movement selections (Shen et al., 2000; Pomplun et al., 2001; Shen and Paré, 2006) and saccade trajectories themselves (Kehoe et al., 2018a,b; Giuricich et al., 2023) are biased for objects that share features with the target. Conversely, during task-free viewing of natural scenes, human (Parkhurst et al., 2002; Parkhurst and Niebur, 2003; Peters et al., 2005; Berg et al., 2009) and monkey (White et al., 2017a) eye movements are disproportionately directed toward areas in the scene with the most salient features. Although feature-guided eye movements are so ubiquitous, surprisingly little research has investigated the cognitive and neural mechanisms that incorporates visual features into impending eye movement programs.

## 2. Feature-guided eye movements

Feature-guided eye movements require several processing steps: the spatial position of potential eye movement targets must be encoded, each potential target must be feature-weighted according to behavioral goals, a winner in the set of potential targets must be selected, and the spatial code of the winner must be converted into movement instructions and sent to the extraocular muscles. All these processing steps are observed in the oculomotor substrates of the primate nervous system.

**Figure 1:**
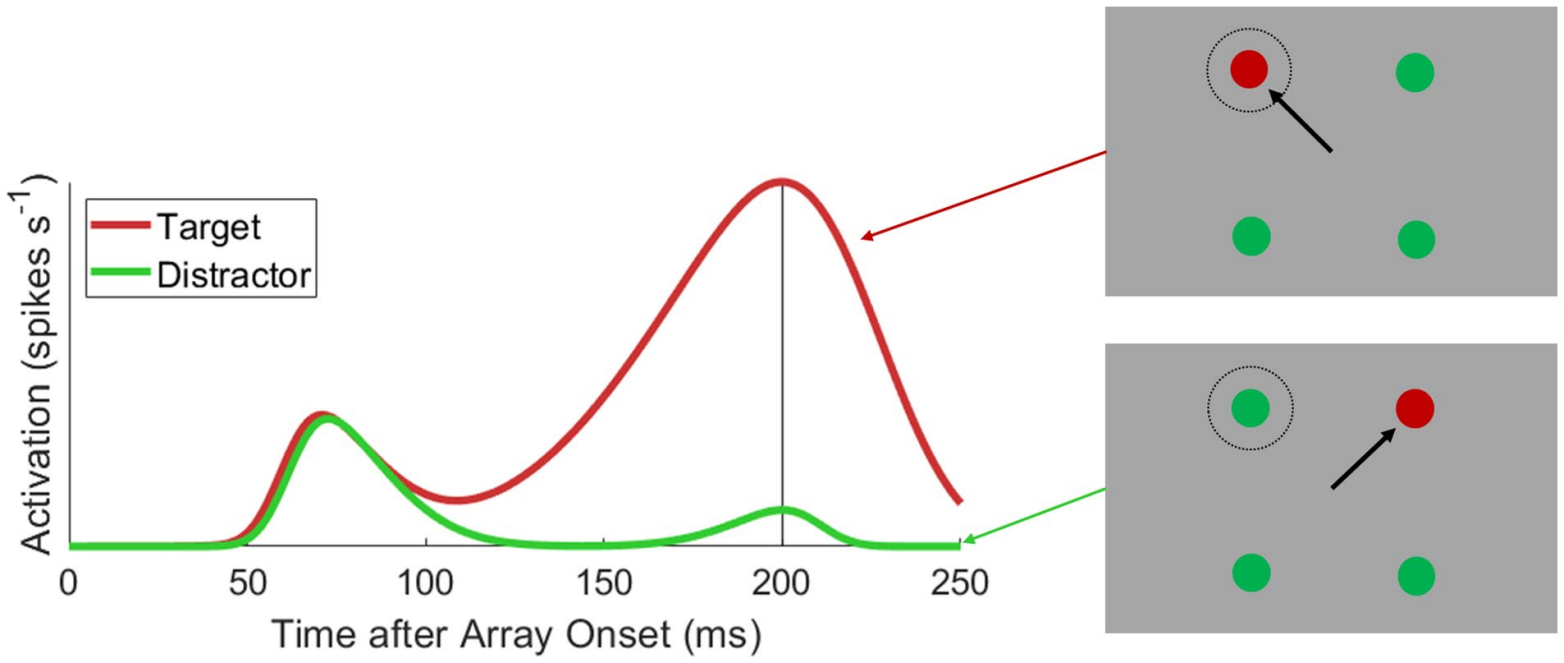
Typical visuomotor neural activation during feature-based target selection. Depicted is a hypothetical color-oddity saccade task (right insets), where monkeys saccade to a red target among green distractors and spikes are collected from a visuomotor cell whose motorfield aligns to the top-left stimulus position in the search array. Activation is plotted as a function of time after search array onset when either a target (red line) or distractor (green line) is placed into the motorfield. There is a feature invariant visual onset burst approximately 50 ms after array onset. After approximately 100 ms, activation encoding targets and distractors diverges (i.e., discrimination time), where target activation ramps up to a motor burst triggering a target-directed saccade and distractor activation decays to baseline.

The primate oculomotor system encodes the loci of visual stimuli and potential eye movements as direction-amplitude vectors on orderly retinotopic motor maps, whereby sufficient activation of a specific vector elicits an eye movement with the corresponding direction and amplitude (Robinson and Fuchs, 1969; Robinson, 1972; Bruce et al., 1985). This is perhaps nowhere more apparent than in the superior colliculus (SC) (Wurtz and Goldberg, 1971, 1972; Sparks, 1978; Mays and Sparks, 1980; Munoz and Wurtz, 1995) and frontal eye fields (FEF) (Goldberg and Bushnell, 1981; Bruce and Goldberg, 1985; Schall et al., 1995a). In these substrates—SC (Horwitz and Newsome, 1999, 2001; Krauzlis and Dill, 2002; McPeek and Keller, 2002; Li and Basso, 2005; Shen and Paré, 2007, 2014; Kim and Basso, 2008) and FEF (Schall and Hanes, 1993; Schall et al., 1995a; Bichot et al., 1996; Thompson et al., 1996; Sato et al., 2001; Sato and Schall, 2003)—the featural identity of visual stimuli can be decoded from the neural activity encoding impending eye movements (see Figure 1). However, this feature encoding unfolds over time. After the onset of a visual stimulus, oculomotor neurons encode the presence of the stimulus with a rapid swell of activation (reviewed by Boehnke and Munoz, 2008). This early activation is feature invariant but is soon reweighted by the feature-based behavioral relevance and conspicuity of the stimulus, typically between 50 and 100 ms after the start of the visual onset burst (reviewed by Fecteau and Munoz, 2006).

Two alternative explanations could account for feature-reweighting of competing incipient oculomotor programs routinely observed in oculomotor research: oculomotor substrates process visual features independently of and in parallel to the perceptual system, or alternatively, potential eye movements encoded in oculomotor substrates could be dynamically feature-weighted by the perceptual system. Although the latter account is widely held—that feature inputs from the perceptual system are pivotal for feature-guided eye movements (*cf.* Fecteau and Munoz, 2006; Schall and Cohen, 2011)—a more detailed theory of the mechanism subserving this process is lacking. What are the factors that determine when feature-reweighting occurs during the time course of oculomotor processing? Where in the perceptual system does this feature information originate? Is this featural mechanism task-dependent or fundamental? These questions have been largely unaddressed.

In this article, we have several goals: (1) to dispel the notion that oculomotor substrates are sufficient for feature-guided eye movements, and to argue instead that feature-reweighting of oculomotor vectors is driven by dynamic input from the perceptual system; (2) summarize recent experiments revealing the feature-dependent time scale of visual encoding in the oculomotor system; (3) based on these experiments, propose a broad theoretical account of the interplay between perceptual and oculomotor systems that facilitates top-down feature-guided eye movements and bottom-up feature encoding in oculomotor substrates more broadly; and (4) lastly, to discuss how the same experimental paradigms used to measure the latency of feature information in the oculomotor system can be used to answer other questions in vision science and possibly even offer a practical diagnostic tool in clinical neuropsychology.

## 3. Oculomotor substrates are insufficient for feature-reweighting

For neural systems to be even theoretically capable of guiding behavior to specific visual features, they must include feature filters that intrinsically encode specific visual features. In this section, we summarize experimental evidence spanning decades that demonstrates that oculomotor substrates depend upon cortical inputs for feature information. In this view, oculomotor substrates contribute to target selection by integrating visual feature information onto spatial movement coordinates. If so, oculomotor and perceptual substrates ought to be functionally dissociable. Therefore, in this section we also summarize the evidence from ablation, inactivation, and microstimulation studies supporting this functional dissociation.

### 3.1. Inherent feature encoding

**Figure 2:**
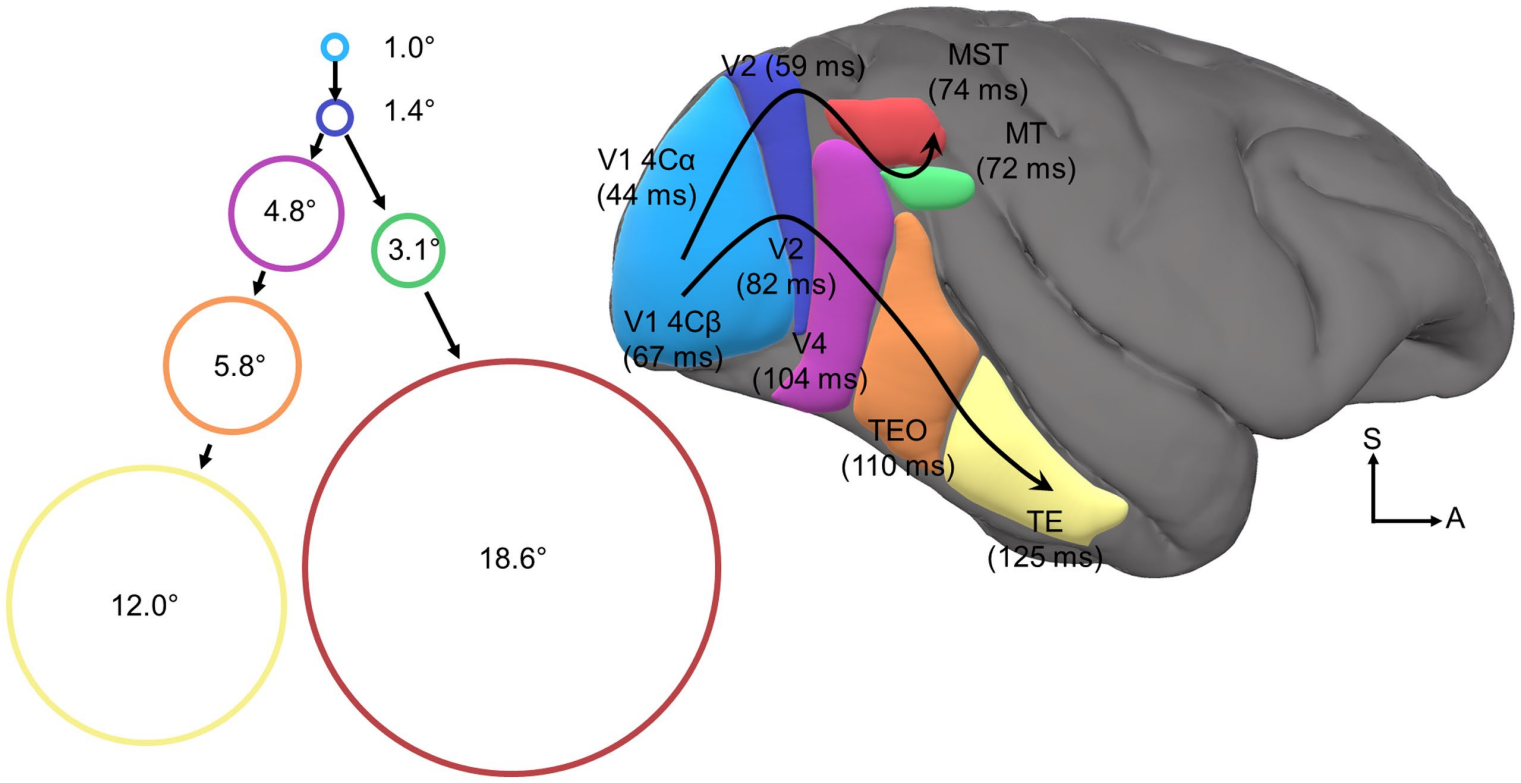
Visual cortical hierarchy. Depicted is a right lateral view of macaque cortex with several color-coded and labeled visual cortical areas. Bracketed values indicate average onset latencies as reported by Schmolesky et al. (1998) for V1/V2/V4/MT/MST and Nowak and Bullier (1997) for TEO/TE. Curved black arrows trace the dorsal and ventral processing streams. Circles are color-coded to indicate visual cortical area and are scaled in size to indicate average visual receptive field area at the fovea as reported by Kravitz et al. (2013) for V1/V2/V4/TEO/TE and Raiguel et al. (1997) for MT/MST. Accompanying receptive field labels specify the receptive field diameter in degrees of visual angle.

The visual perceptual system encodes the size, location, and features of visual stimuli on retinotopic maps widely distributed across a tangled web of cortical modules most famously cataloged by the meticulous work of Van Essen et al. (see Figure 2). These classic neuroanatomy studies of the connections between visual cortical modules have revealed a clear hierarchical organization between modules (Van Essen and Maunsell, 1983; Felleman and Van Essen, 1991; Van Essen et al., 1992), the *cortical visual hierarchy*. The cortical visual hierarchy is primarily organized such that vision is successively projected to increasingly anterior cortical sites along the posterior-to-anterior axis. Intriguingly, the cortical visual hierarchy also exhibits functional hierarchical organization, as the receptive field size, visual onset latency, and representational complexity of visual neurons within modules successively increases at each level of the anatomical hierarchy. Although there is debate about whether the functional-anatomical hierarchical correspondence has meaningful implications for the nature of visual processing more broadly (Bullier and Nowak, 1995; Hegde and Felleman, 2007), the existence of the functional visual hierarchy is undisputed (*cf.* Hegde and Felleman, 2007). Furthermore, the functional hierarchy is the basis for many successful formal models of visual processing (e.g., Riesenhuber and Poggio, 1999, 2000; Serre et al., 2007). As such, we will herein use the term cortical visual hierarchy to describe the anatomical and/or functional hierarchy interchangeably.

In each module of the cortical visual hierarchy, feature filters extract specific visual attributes (e.g., direction, color, orientation). These feature representations are then projected upstream to the next processing stage where they are pooled or transformed into increasingly complex feature representations (Livingstone et al., 2001; Brincat and Connor, 2004, 2006; Mineault et al., 2012; Yau et al., 2013). As neural projections between cortical modules are bound by some conduction velocity, the average visual onset latency of neurons increases somewhat linearly in successively higher stages of the hierarchy (Nowak and Bullier, 1997; Schmolesky et al., 1998). It is important to note that ascribing strict representational feature sets and visual onset latencies to specific cortical modules is an oversimplification, but this scheme does serve as a useful and widely adopted heuristic.

Experimentally detecting inherent feature encoding in oculomotor substrates is convoluted in the context of *target selection*. Target selection refers to the oculomotor behavior whereby a target must be discriminated from distractors and selected for an eye movement. Often, this discrimination is based on visual features (see Figure 1). Critically, whether oculomotor vectors are reweighted by visual features *per se* or simply by behavioral choices is indistinguishable in this context. Single target saccade and passive viewing paradigms circumvents this issue and has indeed revealed several seemingly inherent feature sensitivities in the intermediate (visuomotor) layers of SC: orientation (Chen and Hafed, 2018), spatial frequency (Chen and Hafed, 2017), color (White et al., 2009), motion direction (Davidson and Bender, 1991; Horwitz and Newsome, 2001), and face detection (Nguyen et al., 2014; Le et al., 2020). Similarly, a classic study of FEF visual response properties found that 12% of purely visual FEF neurons show featural sensitivity for color and motion (Mohler et al., 1973), while more recent studies have found that between 31 and 54% of visuomotor FEF neurons exhibit sensitivity for motion direction and speed (Barborica and Ferrera, 2003; Xiao et al., 2006).

A classic experiment by Schiller et al. (1974) provides some insights into the origin of these seemingly inherent feature sensitivities in oculomotor substrates. They demonstrated that the visual feature sensitivities exhibited by SC neurons must be driven by cortical inputs. After the ablation or cortical cooling of striate and extrastriatal cortices, visual responses, but not motor responses, in the intermediate layers of SC were completely extinguished. Conversely, visual responses in the superficial (retinotectal) layers of SC were unaffected. Given the feature sensitivities in SC, it is entirely possible that feature discrimination subserving target selection occurs in SC itself. However, this classic experiment by Schiller et al. makes clear that, even if this is true, SC is dependent on cortical input for feature information. Featural sensitivities in FEF have not been examined following cortical cooling/ablation, so it is unclear whether FEF featural sensitivities also rely on downstream feature-specialized cortical modules. However, is this feasible given that FEF is richly interconnected with visual areas spanning the entire cortical processing hierarchy (Schall et al., 1995b; Barone et al., 2000; Moore and Armstrong, 2003).

The combined weight of evidence from other ablation, inactivation, and microstimulation studies casts further doubt on the possibility that oculomotor substrates SC and FEF are the seat of feature discrimination subserving target selection.

### 3.2. Dissociating oculomotor and perceptual functions

In systems neuroscience, there is a decades-old double-dissociation between perceptual and motor processing systems (Mishkin et al., 1983; Haxby et al., 1991; Ungerleider and Haxby, 1994). In this section, we summarize a similar double-dissociation that exists between the neural modules that subserves the perception of visual stimulus features from the neural modules necessary to program and execute eye movements.

#### 3.2.1. Lesions and inactivation

In humans, lesions of the visual cortices are predominately associated with permanent perceptual deficits but spared motor function. When visual cortical lesions are especially localized, the deficits are amazingly specific; limited to the visual attribute(s) for which the lesioned cortex was specialized. Fascinating examples include achromatopsia and akinetopsia, the inability to perceive color or motion (respectively), reviewed elsewhere (e.g., Zeki, 1991; Heywood and Cowey, 2013). These observations suggest that the cortical visual hierarchy exhaustively encodes the features we perceive and experience (Zeki and Bartels, 1999). In contrast to these perceptual deficits, visual cortical lesions spare motor functions.

Hemianopic patients with acquired scotomas from damage to the geniculostriate pathway can still readily execute saccades *per se*; however, to make visually-guided saccades into their lesioned visual field, they reply upon idiosyncratic compensatory strategies that exploit vision from their intact visual field (Meienberg et al., 1981; Barbur et al., 1988). Similarly, when hemianopic patients make saccades into the intact visual field, peripheral distractors in the lesioned field do not elicit saccadic interference (Walker et al., 2000), as is seen in healthy adults (Edelman and Xu, 2009; Buonocore and McIntosh, 2012; Hafed and Ignashchenkova, 2013).

Ablations of oculomotor substrates are associated with deficits in saccadic production and selection. SC and FEF are reciprocally connected but independently project to the brainstem motor circuitry (Schnyder et al., 1985; Huerta et al., 1986; Moschovakis et al., 1988; Stanton et al., 1988; Sommer and Wurtz, 2004). As such, SC and FEF form parallel pathways necessary for planning and executing eye movements, as ablation of both SC and FEF produces permanent deficits in visually guided saccades (Schiller et al., 1979, 1980), while ablation of a single module spares this function (Mohler and Wurtz, 1977; Schiller, 1977; Schiller et al., 1987; but see Hanes and Wurtz, 2001).

Reversible inactivation of oculomotor substrates impairs perceptual discriminations indicated with saccades (McPeek and Keller, 2004; Monosov et al., 2011) and even for manual button responses or reaching movements. However, these behavioral deficits are likely related to an acquired attentional neglect scotoma and do not necessarily imply a perceptual deficit more broadly. Case studies of human localized SC lesions are exceedingly rare, but results from at least one such human case study does support this reasoning. One patient reportedly did develop visuospatial neglect contralateral to a localized lesion of SC; however, the authors did not report whether the perceptual capabilities of the patient were intact (see Nyffeler et al., 2021).

#### 3.2.2. Microstimulation

Microstimulation reveals the functional encoding scheme of neural populations in a complementary manner to lesion and inactivation studies. If the oculomotor substrates rely upon featural representations in the visual cortical hierarchy to program feature-guided eye movements, then microstimulation of either the visual cortices or oculomotor substrates should bias eye movements to the stimulated feature during target selection. Additionally, microstimulation of the visual cortices should elicit perceptual phenomena.

A fascinating human case study by Lee et al. (2000) reports a variety of visual hallucinations evoked by visual electrocorticographical stimulation across occipital, occipital-parietal, and occipital-temporal cortices. Patients experienced seeing flashes, primitive shapes, formless “blobs,” complex objects including faces and animals, and even entire scenes, where objects were either moving or stationary, and objects appeared in various colors and textures. Schiller et al. (2011) showed that visual perceptual phenomena elicited by microstimulation of monkey visual cortices can be inferred with clever behavioral paradigms, likely inspired by the elegant somatosensory experiments of Romo et al. (1998). Their research suggested that monkeys experienced seeing a small, low-contrast colored dot after microstimulation was delivered to striate cortex.

Eye movements can be elicited from microstimulation of striate cortex, where the current threshold drops as a function of the cortical penetration depth (Tehovnik et al., 2003). This is consistent with much earlier experiments showing that striatially-evoked eye movements arise from current propagating through the direct connection between striate cortex and SC, as these striatally-evoked eye movements are abolished when SC is ablated (Schiller, 1977).

Microstimulation of the oculomotor substrates elicits eye movements at relatively low currents (Robinson and Fuchs, 1969; Robinson, 1972; Bruce et al., 1985). At even lower subthreshold currents, microstimulation of the oculomotor substrates mimics the behavioral and neural effects of visuospatial attentional deployment, namely speeded processing and lowered perceptual detection thresholds (Moore and Fallah, 2001, 2004; Carello and Krauzlis, 2004; Cavanaugh and Wurtz, 2004) and downstream neural gain modulation (Moore and Armstrong, 2003; Armstrong et al., 2006). Similarly, speeded detection times and perceptual biases are elicited from microstimulating visual cortices, such as medial temporal (MT) (Salzman et al., 1990, 1992; Celebrini and Newsome, 1995; Britten and van Wezel, 1998; DeAngelis et al., 1998; Bisley et al., 2001; Ditterich et al., 2003) and V4 (Kienitz et al., 2022). Critically, these perceptual biases are incorporated into eye movements, as pursuit eye movements intended to track a moving stimulus are biased in the cortically (i.e., MT) microstimulated movement direction (Komatsu and Wurtz, 1989; Groh et al., 1997; Born et al., 2000).

Taken together, these results demonstrate that oculomotor substrates lack cortically-independent inherent feature representations, while inactivation and stimulation studies show that they functionally subserve attentional selection and eye movement generation, but not perception. Conversely, the cortical visual hierarchy is the seat of visual feature-encoding in the nervous system: inactivation and stimulation studies show that visual cortices are necessary for the perception of visual features, and critically, these feature representations bias feature-guided oculomotor behavior.

## 4. Feature dependent visual onset latencies

A basic property of the cortical visual hierarchy is that increased featural complexity requires additional featural processing in higher stages of the hierarchy and thus additional processing time. The behavioral consequence of this encoding scheme is that the time required to perceive features is proportional to their complexity (Bodelón et al., 2007). Similarly then, if oculomotor vectors are dynamically feature-reweighted by inputs from the perceptual system, then increasing the featural complexity of a potential eye movement target should increase the latency of its feature-reweighing.

**Figure 3:**
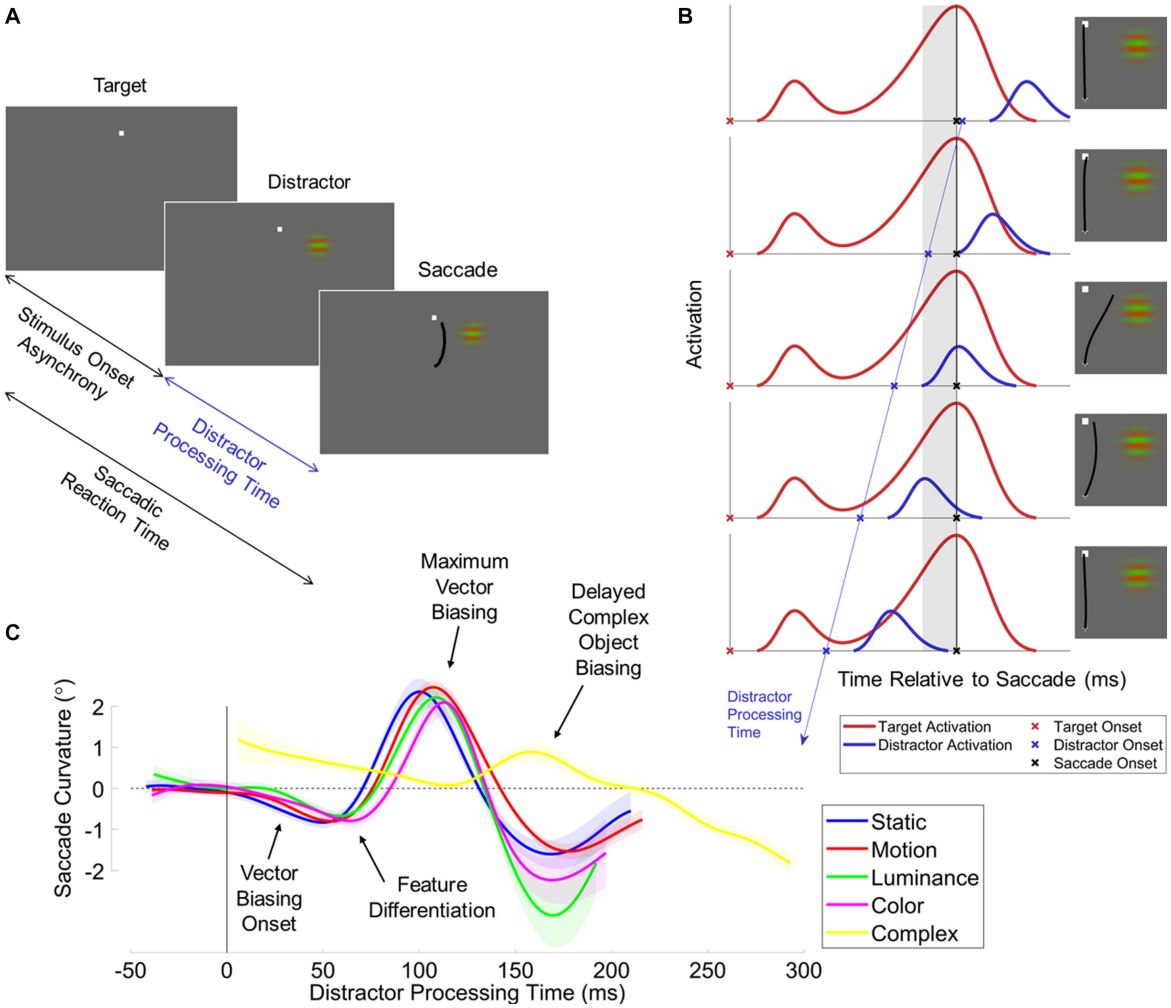
The distractor-saccade onset asynchrony (SDOA) paradigm. **(A)** Task procedure schematic. Targets are displayed at some random interval before the distractor (stimulus onset asynchrony). Participants elicit target-directed saccades sometime after distractor onset (distractor processing time). **(B)** Putative neural underpinnings. Depicted is the hypothetical activation of visuomotor neurons elicited when the target (red line) or distractor (blue line) are in the motorfield of the cell. Activation is plotted as a function of time relative to the saccade. The onset of the saccade is indicated by the black vertical line and black “X”s. In the perisaccadic interval immediately prior to the saccade (gray shaded region), the incipient motor plan for target-directed saccades may become biased by unresolved distractor activation. The target is displayed sometime prior to the saccade (red “X”s). In this contrived example, the saccadic reaction time is constant. The target onset elicits a visual onset burst time-locked to stimulus onset and a motor burst time-locked to the saccade. The distractor onset (blue “X”s) is stochastic and can occur at any time relative to the saccade. The distractor onset elicits a visual onset burst time-locked to stimulus onset. In this example, we systematically increase the distractor processing time (i.e., the duration of time between distractor onset and subsequent saccade), illustrated by the blue line with arrowhead. As such, the distractor-related visual onset burst sweeps through the perisaccadic interval. Right insets depict hypothetical saccades elicited by the distractor visual onset burst given its position in time relative to the perisaccadic interval. **(C)** The results across iterations of the SDOA paradigm. Saccade curvature (vector biasing) is plotted as a function of distractor processing time for all examined distractor features: static-gratings (blue), motion-gratings (red) (Kehoe et al., 2023); luminance-modulated Gabors (green), color-modulated Gabors (magenta) (Kehoe and Fallah, 2017); and complex pseudo-alphanumeric characters during discrimination (yellow) (Kehoe et al., 2021). We highlight notable effects with text-labeled arrows: (1) for simple gratings, vector biasing onsets after just 25 ms of distractor processing time; (2) features begin to differentiate after approximately 50 ms of distractor processing time; (3) the maximum vector biasing occurs after approximately 100 ms of distractor processing time; and (4) discriminated complex objects elicit vector biasing very late, after approximately 110 ms. Note that these data from prior publications have been replotted onto a common figure.

Guided by this logic, we have recently conducted a series of behavioral experiments in which we (1) non-invasively infer the oculomotor encoding time course of a visual stimulus and (2) compare this time course between different visual features that constitute the visual stimulus (see Figure 3).

In our paradigm (Kehoe and Fallah, 2017; Kehoe et al., 2021, 2023), human participants plan and execute a saccade to a target, and at some randomized interval after the onset of the target, we onset a peripheral distractor (see Figure 3A). Critically, we constrain the randomized interval between target and distractor onset so to maximize the likelihood that the distractor onsets prior to the saccade. As such, we have referred to this behavioral paradigm as the saccade-distractor onset asynchrony (SDOA) paradigm. Here, we will subsequently refer to the interval between distractor onset and saccade initiation as distractor processing time, as it corresponds to the duration of time afforded to visual processing of the distractor. We then measure a battery of human saccade metrics to examine the effect of the distractor on the target-directed saccade across a continuous range of distractor processing times.

Several recurring patterns of behavior were observed using this paradigm (see Figure 3C): (1) at the shortest distractor processing times, the distractor has no discernable effect on the target-directed saccade; (2) after approximately 25 ms of distractor processing time, saccade trajectories and endpoints curved toward the distractor; (3) by approximately 50 ms of distractor processing time, we saw the onset of a transient drop in the likelihood of generating a saccade; and (4) by approximately 100 ms of distractor processing time, trajectory spatial biasing and the drop in saccade likelihood both reached their maximum extent. These results are generally consistent with other behavioral paradigms that utilize an intervening stimulus during saccade planning to a target (Reingold and Stampe, 2002; Edelman and Xu, 2009; Buonocore and McIntosh, 2012; Hafed and Ignashchenkova, 2013) or double-stepping targets (Becker and Jürgens, 1979; Findlay and Harris, 1984).

The most clear and parsimonious interpretation of these behavioral results is that, in oculomotor substrates like SC, the visual onset burst encoding the distractor was spatially averaged with the developing target-directed saccade program (see Figure 3B). First, visual onset bursts observed in oculomotor substrates generally occur with a latency of ~50 ms after stimulus onset (Boehnke and Munoz, 2008), consistent with a broad set of behavioral results. Second, invasive microstimulation (Glimcher and Sparks, 1993; McPeek et al., 2003; McPeek, 2006) and dual recordings (Port and Wurtz, 2003) have confirmed that unresolved distractor activation does indeed spatially bias saccades according to a vector average of the target and distractor vectors.

### 4.1. Luminance and color

The most intriguing results from our SDOA paradigm arose when we compared the saccadic perturbation (i.e., spatial biasing and drop in saccadic likelihood) time course between different distractor visual features. In the original implementation (Kehoe and Fallah, 2017), we compared luminance- and color-modulated Gabors and observed that saccade curvature elicited by color lagged behind saccadic curvature elicited by luminance (see Figure 3C). The lag was approximately 20 ms, consistent with a neurophysiological study measuring a 30 ms difference between the visual onset bursts of collicular neurons encoding similar visual stimuli (White et al., 2009). Given this close correspondence, we interpreted our result through the lens of the same underlying neural mechanism.

A 20–30 ms lag between color and luminance encoding is consistent with the visual onset latency differences observed in the magnocellular and parvocellular layers of the lateral geniculate nucleus (LGN), between layers 4B/4Cα and 4Cβ in V1, or between the thick and pale thin layers of V2 (Nowak et al., 1995; Schmolesky et al., 1998). The anatomical divisions in these early visual substrates are highly functionally segregated and form parallel processing streams of disjoint feature sets (reviewed by Livingstone and Hubel, 1988), including luminance and color in particular. This experiment therefore confirms our reasoning: specific visual features should elicit visual onset latencies associated with specific visual processing modules specialized for processing those features. As such, the results of White et al. (2009) and Kehoe and Fallah (2017) taken together strongly imply that the representations of potential eye movement targets in oculomotor substrates are dynamically feature-weighted by the relevant modules in the perceptual system.

### 4.2. Non-motion and motion

The results of Kehoe and Fallah (2017) suggest that feature information is projected into the oculomotor system from the relevant processing *channels* of the cortical visual hierarchy. But it remained difficult to determine whether feature information is projected into the oculomotor system from the relevant *stage* of processing in the cortical visual hierarchy. We therefore recently repeated the original paradigm replacing the distractor features with static and motion-animated gratings (Kehoe et al., 2023). Unlike luminance and color information, which are processed in parallel channels within the same cortices and subcortical nuclei, grayscale static and motion stimuli are encoded within the same processing channel, which is distributed between and within cortices. For example, V1 encodes simple motion components and projects these upstream to MT where they are summated into complex motion representations (Movshon and Newsome, 1996). In V1, direction-selective complex cells encode motion direction by spatiotemporally summating input from phase-selective simple cells (De Valois and Cottaris, 1998). Therefore, by using a grayscale static and motion feature set in the SDOA paradigm, any oculomotor encoding delays between features would suggest that feature information projected into the oculomotor system was bottlenecked to accommodate the appropriate stage of cortical feature processing.

In this experiment (Kehoe et al., 2023), we made several interesting findings: (1) at the earliest distractor processing times (<50 ms), saccadic perturbation was feature invariant; (2) after 50 ms of distractor processing time, saccadic perturbation elicited by motion gratings lagged behind saccadic perturbation elicited by static gratings by 10 ms; and (3) this temporal lag was not accompanied by any saccadic perturbation magnitude differences between features (see Figure 3C).

The biasing of saccade trajectories observed for distractor processing times between 25 and 50 ms suggests that our behavioral paradigm is sensitive to oculomotor processing occurring at the theoretical lower bound of visual afferent latencies. The direct connections from the retina to the superior colliculus—constituting a relatively small number of retinal ganglian projections—is the only known mechanism to support these conduction latencies (Hubel et al., 1975; Schiller and Malpeli, 1977). Visual onset bursts in collicular neurons are not typically seen at latencies less than ~50 ms after visual stimulus onset (Boehnke and Munoz, 2008). These results may therefore suggest a gentle increase of baseline collicular activity that directly upregulated upstream brainstem oculomotor nuclei in a passthrough manner, but this speculation warrants investigation.

The saccade trajectory biasing time course became differentiated between static and motion distractors after 50 ms, where the effects of motion distractors lagged those of static distractors by 10 ms. Similarly, a rapid drop in saccadic likelihood began at 50 ms for static distractors and later at 60 ms for motion distractors. Saccadic inhibition immediately following visual stimulation is likely due to rapid lateral inhibition networks in colliculus, whereby activation of a saccade vector near instantaneously inhibits neighboring saccade vectors (Munoz and Istvan, 1998). This is suggested by the facts that (1) visual onsets elicit transient collicular bursts and visually-evoked saccadic inhibition is also transient and (2) visual onset bursts and saccadic inhibition occur after the same latency (Reingold and Stampe, 2002; Edelman and Xu, 2009; Buonocore and McIntosh, 2012; Hafed and Ignashchenkova, 2013). Given that the feature-dependent saccade inhibition effect was very likely driven by collicular visual onset bursts encoding the distractor, then by extension, the trajectory biasing divergence occurring simultaneously was likely then also driven by collicular visual onset bursts.

The feature-dependent saccade perturbation latencies we saw further corroborates our account that visual feature information projected into the oculomotor system is bottlenecked to afford the requisite processing in the appropriate substrates of the cortical visual hierarchy. Furthermore, we did not see any differences in the magnitude of saccadic vector averaging between features. Therefore, it is unlikely that the time course erroneously appeared differentiated because the motion burst continued to intensify after the static burst reached its maximum intensity.

### 4.3. Complex object discrimination

The most compelling evidence of bottlenecked visual projections into the oculomotor system was when we examined the oculomotor encoding time course of complex, novel objects during a discrimination task (Kehoe et al., 2021). On this task, participants were shown a target preview and told to discriminate this target from a distractor with a saccade to indicate their choice. These stimuli resembled pseudo-alphanumeric characters that were not meaningful to English speakers. As in other iterations of our behavioral paradigm, we randomized the stimulus onset asynchrony between targets and distractors. However, to ensure that stimulus order did not provide reliable target information, the distractor onset prior to the target on 50% of trials. We were able to analyze distractor processing time as before by concentrating our analyses on trials with targets leading distractors and distractors leading saccades. We were therefore able to measure saccade trajectory biases, saccadic inhibition, and error rates as a function of distractor processing time.

Fascinatingly, we observed that the earliest evidence of trajectory biasing, saccade inhibition, and selection errors was at distractor processing times of at least 110 ms, in stark contrast to the 50 ms effects we saw for simple, task-irrelevant gratings. The discrimination of these stimuli would very likely recruit substrates in the higher stages of the cortical processing hierarchy, specifically inferotemporal cortex (IT) where simple geometric subunits represented in downstream modules are concatenated into coherent objects (Brincat and Connor, 2004, 2006). At these later stages in the hierarchy, visual onset latencies are typically over 100 ms (Nowak and Bullier, 1997). As such, the clearest explanation of our results is that visual encoding of the complex objects was absent within the oculomotor substrates until these objects were visually represented in the higher stages of the cortical visual hierarchy, the necessary substrates for complex object discrimination.

## 5. Mechanism for feature representations in oculomotor substrates

Our experiments suggest that visual encoding in the oculomotor system is extremely contextual. A combination of the task requirements and the visual feature set determines the latency of visual encoding in the oculomotor system. Since the oculomotor system relies upon cortical input for visual feature information (Schiller et al., 1974), we argue that these latency differences reflect the highest stage of processing in the cortical visual hierarchy that was recruited in each experimental context to successfully satisfy feature discrimination. In this view, only after visual representations have manifested in these task- and feature-dependent cortical substrates does the oculomotor system receive cortical inputs to reweight eye movement vectors. As there are systematic differences between the visual onset latencies across the cortical visual hierarchy, the onset latency of visual representations in the oculomotor system is also feature-dependent and increases with featural complexity. This framework is inspired by classic cognitive theories stipulating that a *base representation* (Ullman, 1984) or *raw primal sketch* (Marr, 1982) must be constructed before cognitive mechanisms or *visual routines* (Ullman, 1984) can operate on the visual information to satisfy relevant visual task demands.

Neurophysiological investigations of saccadic target selection typically utilize discriminations between different features of the same visual attribute (e.g., a red target among green distractors, where all stimuli are color singletons). In these experiments, oculomotor visual onset bursts for targets and distractors have identical latencies. Shortly thereafter however, the activation level of the target gradually increases, while the activation level of distractors gradually decreases (Fecteau and Munoz, 2006). These observations suggest that, after visual input first arrives in the oculomotor substrates, feature-dependent cortical inputs must continue to dynamically reweight the saccade vector over time. This likely reflects the fact that in the recruited cortical modules, visual discriminations unfold over time by way of gradually shifting neuronal feature representations, akin to the process Ullman (1984) has termed *incremental representations*. When observers encounter stimuli sharing just one attribute (e.g., all stimuli are color singletons), then the same cortical module(s) are recruited to encode all visual stimuli. Thus, the cortical bottleneck applies equally to all stimuli in this context. We therefore do not expect a visual onset burst latency difference between stimuli, as is seen. After the feature-dependent cortical modules are recruited for the task and begin representing the visual stimuli, the cortical feature discrimination process begins. Once commenced, these relevant cortical module(s) dynamically reweight the oculomotor vectors to maintain parity between oculomotor and cortical feature representations. Thus, we observe target features activate and distractor features deactivate over time in oculomotor substrates during target selection.

Another critical implication from our behavioral work is that this putative neural mechanism is not specifically a mechanism for feature *discrimination* in oculomotor substrates, but more broadly feature *representation* in oculomotor substrates. In two of our experiments (Kehoe and Fallah, 2017; Kehoe et al., 2023), we observed saccadic perturbation latency differences between distractor features that were wholly task-irrelevant, as these distractors always appeared at target invalid spatial locations. Feature discriminating these targets from distractors was not necessary for the task and discrimination could have been achieved more simply with spatial processing. Despite this, we still observed that saccadic perturbation latencies were contingent on featural complexity. This suggests that our putative neural mechanism does not just subserve feature-based oculomotor target selection but instead describes a fundamental processing regime connecting the oculomotor and perceptual systems.

## 6. Caveats and alternatives

### 6.1. Categorizing neural substrates

In this review, we have focused on SC and FEF as the critical substrates of the oculomotor system. However, these substrates are just two of many substrates widely considered part of a broad oculomotor network (Corbetta et al., 1998; Fecteau and Munoz, 2006; Schall and Cohen, 2011). For example, the lateral bank of the intraparietal sulcus (LIP) is widely considered a critical substrate subserving saccadic behavior (Andersen et al., 1992; Goldberg et al., 2006) because, like in SC and FEF, it encodes both spatial and feature information during saccadic target selection (Constantinidis and Steinmetz, 2001; Buschman and Miller, 2007; Subramanian and Colby, 2014), lesions to LIP produce attentional neglect scotomas (Driver and Mattingley, 1998; Parton et al., 2004), eye movements are evoked from weak microstimulation (Tehovnik et al., 2003), and it exhibits perisaccadic receptive field remapping (Duhamel et al., 1992). However, these properties are not unique to LIP and are observable, to at least some extent, in several clearly visual cortices such as V1 [task-modulated feature discriminability (Motter, 1993; Chen and Seidemann, 2012), post-lesion scotomas (Weiskrantz, 1996), evoked saccades (Tehovnik et al., 2003), perisaccadic receptive field remapping (Nakamura and Colby, 2002; Merriam et al., 2007)]. What does seem to be unique about SC and FEF—and why we mainly focus on these substrates when discussing critical oculomotor substrates here—is that they are directly connected to the brainstem pulse generators (Schiller and Tehovnik, 2005).

In a complimentary manner, the question arises whether SC and/or FEF are *visual* areas. There are compelling reasons to draw this conclusion, as recently summarized by Hafed et al. (2023) concerning SC in particular. For example, the optic tectum phylogenetically precedes visual cortex altogether and is the primary mechanism for vision in some organisms. As summarized previously, there has recently emerged a broad understanding of the rich feature processing capabilities of the SC. Finally, SC is richly interconnected with most of cortex and subcortex linking it to structures specialized for visual and cognitive processing. Granting SC as a visual area given these interesting considerations, SC is certainly still an oculomotor substrate in primates given its privileged synaptic proximity to the brainstem, as discussed above. As such, although there is a growing appreciation for SC as a both a visual and oculomotor substrate, it is, nevertheless, still oculomotor.

### 6.2. Interpreting absolute latencies

Several studies have specifically examined the latency of visual onsets in oculomotor areas during passive free-viewing (Mohler et al., 1973; Schall, 1991; Schmolesky et al., 1998; Pouget et al., 2005; Mayo and Sommer, 2013). Comparing the average visual onset latencies in oculomotor substrates (typically 50–60 ms) observed in these studies to the average visual onset latencies observed across the cortical visual hierarchy in other studies (see Nowak and Bullier, 1997) seems to suggest that visual onset latencies in oculomotor substrates are faster than those observed in many modules of the cortical visual hierarchy. However, this is a complex comparison to make as latencies are inherently variable; sensitive to individual differences between organisms, states (e.g., anesthetized vs. awake), tasks, and myriad stimulus parameters (e.g., contrast, size, position).

Circumventing this issue, one neuronal chronometry study by Schmolesky et al. (1998) recorded visual onset latencies across cortical modules—mostly visual but also including FEF—within the same anesthetized monkeys. They observed that there was no difference in the average visual onset time between FEF neurons and neurons in higher areas of the dorsal cortical processing stream, namely areas V3, MT, and MST. Additionally, visual onset latencies were actually faster in FEF than in the pale thin layers of V2 and in V4. However, it is not clear from this study whether this pattern of results is specific to the task and stimulus set that was used. For example, in the context of planning a saccade to a single high contrast spot of light, why would FEF wait for visual input from V4 when visual input from LGN or V1 is likely sufficient to provide FEF with the necessary visual information? Indeed, our own behavioral experiments suggest that oculomotor visual onset latencies are stimulus-dependent, so it is entirely possible that FEF visual responses are slower than those in V4 for some other task/stimulus set.

Yet another possibility is that visual onset latencies are absolute and the mechanism that reweights oculomotor vectors based on features is by virtue of cascading visual input into oculomotor substrates. As SC and FEF are reciprocally innervated by nearly the entire visual brain, perhaps visual input from across all nodes of the visual cortical hierarchy is projected into the oculomotor substrates sequentially. For example, perhaps visual responses in FEF are initially driven by inputs from V1, then driven by V2 10 ms later, thus visual onset latencies in FEF are faster than in V2. This also is consistent with previous neuronal chronometry experiments showing a very wide range of visual onset latencies between cells in oculomotor substrates (30–120 ms) (Mohler et al., 1973; Schall, 1991; Schmolesky et al., 1998; Pouget et al., 2005; Mayo and Sommer, 2013) similarly observed across modules of the cortical visual hierarchy (Nowak and Bullier, 1997). As such, the simplest explanation linking these observations could be that the oculomotor visual response latencies are driven by the fastest early sensory responses.

Another important consideration is that examining the onset of feature sensitivities as opposed to visual onset latencies likely provides a better indication of when feature information arrives in oculomotor substrates. As discussed, oculomotor stimulus encoding evident in neural spikes is usually feature invariant for the initial ~100 ms after stimulus onset (Boehnke and Munoz, 2008). Our own behavioral experiment shows that stimulus information is decodable from saccade metrics in as little as 25 ms, and unsurprisingly, is also feature invariant (Kehoe et al., 2023). If this initial visual encoding is entirely spatial (see Fecteau and Munoz, 2006), then it is the wrong metric to compare the latency of feature information between visual and oculomotor modules. For example, White et al. (2009) compared SC visual onset latencies evoked by isoluminant color targets to those evoked by luminance targets on a simple saccade-to-target task. They observed that visual onsets for color targets lagged luminance targets by at least 30 ms. Critically, however, they also observed that the color responses exhibited tuning in DKL colorspace and thus necessarily conveyed feature information and not merely spatial information. As such, this is a very robust comparison of feature information latency differences and provides an extremely useful example of how to easily test our theoretical account posited here.

### 6.3. Reciprocal processing

We argued strongly that feature information manifests in oculomotor substrates only after antecedent featural processing in cortical substrates. However, a number of studies suggest that this relationship is far more reciprocal than has been outlined here. In a seminal experiment, Moore and Armstrong (2003) microstimulated FEF while also recording from downstream neurons in V4 with overlapping or non-overlapping receptive fields. They observed that neuronal visual activity in V4 was enhanced by stimulation of retinotopically congruent loci in FEF and was suppressed by stimulation of retinotopically incongruent loci. In a complimentary experiment, showed that inactivation of FEF increased presaccadic enhancement of V4 activity and decreased feature-based discriminability of V4 visual responses. That is, V4 began strongly encoding the direction of saccades and exhibited a reduced sensitivity to encode the features of visual stimuli.

These observations clearly and elegantly demonstrate that feature representations in visual cortices are modulated by reciprocal feedback from upstream oculomotor substrates. What is less clear from these experiments is whether the modulation of downstream sensory representations is feature-based or purely spatial. Afterall, microstimulating FEF also produces behavioral effects akin to exogenously cueing spatial attention (Moore and Fallah, 2001, 2004), so FEF modulation of V4 feature representations in these experiments can be accounted for by spatial processing and may be entirely unrelated to feature processing in FEF.

Other experiments have provided evidence of feature processing in oculomotor substrates manifesting earlier than in select visual cortices. showed that feature discrimination occurs in FEF 30–50 ms before it occurs in V4 during cued visual search for complex objects. Similarly, White et al. (2017b) recently showed that task-irrelevant salience (i.e., orientation contrast) is encoded in SC approximately 10 ms earlier than in V1 during a simple saccade-to-target task. These observations raise several potential explanations.

First, it is entirely possible that the choice of visual modules on these tasks were higher in the visual hierarchy than was sufficient to discriminate the stimuli. That is, perhaps if an earlier module was recorded from, feature discrimination would occur earlier in the visual module than in the oculomotor module. For example, substituting V2 for V4 in the case of LGN with V1 in the case of White et al. (2017b). This possibility cannot yet be ruled out but poses a difficult experimental challenge. Second, perhaps a primary function of oculomotor substrates is as a comparator. In the case of SC, previous authors have long argued that its function is to agnostically pool feature representations from sensory cortices and compute salience based on disparate feature codes (Fecteau and Munoz, 2006; White et al., 2017a,b). In the case of FEF, there is also strong evidence of salience encoding (Sato and Schall, 2003; Thompson and Bichot, 2005). In this comparator view, perhaps feature discrimination is delayed in sensory cortices relative to oculomotor modules because feature discrimination in sensory cortices is delayed until salience information is reciprocally propagated by oculomotor substrates back into sensory cortices.

Clearly, reciprocal interactions between oculomotor substrates and sensory cortices are well-supported experimentally. However, reciprocal sensorimotor interactions are not mutually exclusive with the theory posited here. Future investigations could examine potential latency differences between stimulus attribute types (e.g., luminance vs. color) and may discover that although feature discrimination in oculomotor substrates does precede feature discrimination in visual cortices (e.g., FEF before V4), discrimination for more complex features occurs later than simpler features within those oculomotor substrates.

## 7. Extensions of the SDOA paradigm

The SDOA paradigm can be used to answer a broad range of questions in vision and cognitive science and has many clinical applications. Inferring stages of processing has been a central theme of vision and cognitive science throughout the entire contemporary period (e.g., Neisser, 1967; Treisman and Gelade, 1980; Marr, 1982; Ullman, 1984). Our paradigm offers a robust tool to assess the processing stage of a stimulus across time, but its applicability is also not limited to inferences of feedforward visual processing in the cortical visual hierarchy. The advantage of examining behavior, as with the SDOA paradigm, is that it reflects the output of the entire cognitive information processing pipeline, including executive, memory, sensory, and affective subsystems. Critically, the output of these various subsystems is encoded by eye movements (Takikawa et al., 2002; Theeuwes et al., 2005; Belopolsky and Theeuwes, 2011; Schmidt et al., 2012) and neural activation within oculomotor substrates (Hanes et al., 1998; Ikeda and Hikosaka, 2003; Paré and Hanes, 2003; Johnston and Everling, 2006, 2008). As such, the SDOA paradigm examines how the output of these various cognitive subsystems is encoded into oculomotor programs over time.

There is a clinical tradition of using eye movements for early diagnosis of neurological disease and abnormality (for review, see Anderson and MacAskill, 2013; Antoniades and Kennard, 2015), as eye movement tasks are quick, non-invasive, computationally light, and inexpensive to administer. However, although abnormal eye movements are indicative of neurological disorders, they do not differentiate between neurological disorders. The SDOA paradigm examines eye movements across stages of processing and can selectively focus on specific cognitive subsystems. Therefore, our paradigm lends itself to differential and more sensitive diagnosis. Furthermore, the SDOA paradigm is an effective means to trace disease or rehabilitative progress, without the need for more difficult, costly, and invasive medical surveillance methods.

## 8. Conclusion

Visual features often guide primate eye movements (Shen et al., 2000; Pomplun et al., 2001; Shen and Paré, 2006) and can be decoded from neural activation encoding potential eye movements (Boehnke and Munoz, 2008). However, oculomotor research has overlooked the mechanism that feature-reweights potential eye movements. Here, we have summarized functional and anatomical evidence that strongly suggests the oculomotor system is insufficient to extract visual features that guide target selection, and instead, relies upon the substrates of the cortical visual hierarchy for feature information. The cortical visual hierarchy is functionally organized (Felleman and Van Essen, 1991) such that specific visual feature sets are represented in specific modules. Similarly, the onset latency of vision is systematically different between these modules (Nowak and Bullier, 1997; Schmolesky et al., 1998). As such, our account of oculomotor feature-reweighting predicts that feature information should manifest in the oculomotor system with the same latency as in the relevant cortical modules specialized for processing the respective features. Consistent with this prediction, we have conducted a series of innovative behavioral experiments showing that visual features manifest in the oculomotor system in order of visual complexity regardless of whether the features are task-relevant. We therefore proposed a theory of oculomotor feature-reweighting whereby visual feature sets engage a specific set of cortical modules and visual projections into the oculomotor system are delayed until after these cortical modules generate visual representations. During the process of feature discrimination in the recruited cortical module(s), the evolving feature representations are projected to oculomotor substrates where they continuously and dynamically reweight the active eye movement vectors. This theory accounts for many observations in oculomotor research, offers a more detailed account of how oculomotor vectors are feature-reweighted during target selection, and makes a series of easily testable predictions. Finally, we briefly discussed the applicability of the SDOA paradigm to address broader questions in vision and cognitive science and its potential utility as a clinical diagnostic tool.

## Data availability statement

The original contributions presented in the study are included in the article/supplementary material, further inquiries can be directed to the corresponding author.

## Author contributions

DK drafted the manuscript and prepared the figures. DK and MF revised the manuscript. MF approved final draft of the manuscript. All authors contributed to the article and approved the submitted version.
